# Heterogeneity of Organoclay Complexes in Shale Regulates the Generation of Shale Oil

**DOI:** 10.1021/acsomega.4c02101

**Published:** 2024-07-05

**Authors:** Jingong Cai, Jiazong Du, Longfei Lu, Qigui Jiang, Xiaoxiao Ma, Jinyi He

**Affiliations:** † State Key Laboratory of Shale Oil and Gas Enrichment Mechanisms and Effective Development, Beijing 100101, China; ‡ School of Ocean and Earth Science, Tongji University, Shanghai 200092, China; § Key Laboratory of Submarine Geoscience and Prospecting Technology, College of Marine Geoscience, Ocean University of China, Qingdao 266100, China; ∥ Wuxi Research Institute of Petroleum Geology, Sinopec Exploration and Production Research Institute, Wuxi 214126, China

## Abstract

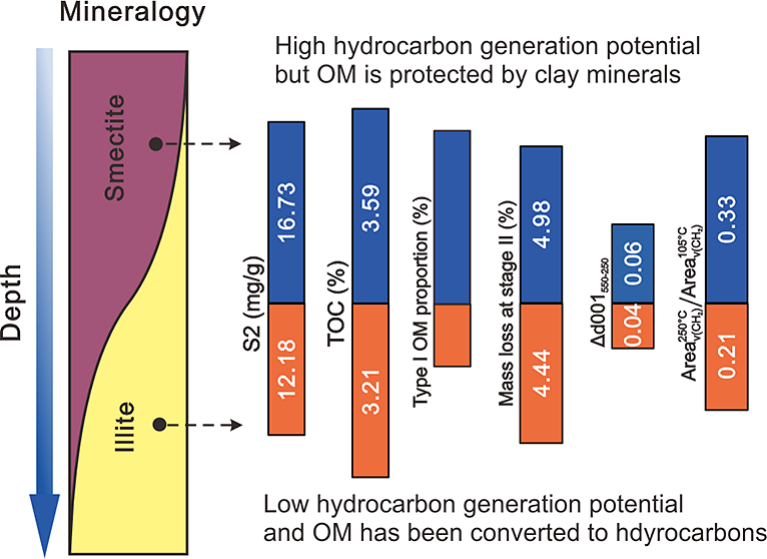

Organic matter (OM) and clay minerals are important components in shale, which are intimately associated with each other in the form of organoclay complexes. The diverse mineral-OM associations result in varying OM occurrences, which possess distinct hydrocarbon generation potential and ultimately affect the accumulation of shale oil. Therefore, the investigation of the heterogeneity of organoclay complexes is crucial to gaining a comprehensive understanding of the varying exploration potential of shale oil resources. In the present study, shale samples from three intervals in Dongying Depression were collected to investigate the mineralogical and organic characteristics of the organoclay complexes, aiming to explore their impact on the yield and composition of shale oil. Results showed that the smectite gradually converted into illite, which was accompanied by the release of OM from clay mineral interlayers and the desorption of chemically adsorbed OM. The yield and composition of shale oil cannot solely be explained by the OM content and types in the shale. Instead, they are intricately linked to the evolution of minerals and OM occurrence. From the perspective of the heterogeneity of organoclay complexes, despite the abundant OM content in shallower intervals (Es3x), the shale oil formation remains limited due to the low degree of mineral evolution and the stabilization of the adsorbed OM by clay minerals. Consequently, this leads to a higher proportion of resin, which is not conducive to the mobility of shale oil. In contrast, despite the OM content varying slightly in the deeper interval (Es4s), the elevated smectite illitization degree promotes the desorption of OM and its conversion into hydrocarbons. This results in a substantial increase in shale oil formation and a higher proportion of saturates, greatly enhancing the mobility of shale oil. These findings are profoundly significant for understanding shale oil generation and accumulation.

## 1 Introduction

The continental oil shale, which is characterized by the extensive distribution and considerable vertical thickness, holds immense potential for shale oil exploration and is poised to be the successor to conventional petroleum resources. However, the exploration practice has uncovered significant variations in the types of “sweet spots” present^1^ and the maturity of shale oil spans a broad range from low to medium-high.^2,3^ The strong heterogeneity of continental shale oil poses significant challenges in predicting prospective “sweet spots,” ultimately constraining the efficient exploration and exploitation of shale oil.

Previous studies have shown that the oil-bearing property of shale is influenced by multiple factors, including organic matter (OM) abundance, maturity, mineral composition, pore structure, and lithofacies.^4−8^ Minerals and OM, as the fundamental components of shale, are intimately associated with each other in the form of organoclay complexes. The organoclay complexes refer to the nanocomposites that are formed by the association between OM and the clay mineral surface at the nanometer scale through various mechanisms (e.g., ion exchange, adsorption, and intercalation).^9,10^ The specific surface area (SSA) of minerals and OM content are usually positively correlated. Approximately 90% of OM in the sediments is preserved on the mineral surface.^11^ For example, in the Cretaceous black shale, more than 85% of the variation in the OM content can be explained by the mineral surface area.^12^ Among the minerals, clay minerals are regarded as the main contributor to OM adsorption primarily due to their extensive SSA, accounting for a remarkable 80% of OM adsorption in shale.^13^ It can be inferred that the mineral-OM interactions are pivotal in determining the enrichment of OM in the shale. However, the composition of minerals and OM in shale varies significantly across different sedimentary environments.^14−16^ These variations would alter the properties of both minerals and OM, such as morphologies, reactivities, and surface structures, resulting in the different mineral-OM associations in shale.^17,18^ Therefore, the OM within organoclay complexes exhibits diverse occurrence.^19^ The OM could be preserved in the pores between grains,^20,21^ physically aggregated with mineral,^22,23^ and chemically adsorbed on the surface of clay minerals or intercalated into the interlayers.^12,24−26^ Physically aggregated OM is generally solvent-extractable, while chemically adsorbed OM forms bonds with active sites of minerals (e.g., coordinately unsaturated Al^3+^). These bonds cannot be disrupted by solvents; thus, the chemically bound OM is not solvent-extractable.

Furthermore, during diagenesis, the types and structures of minerals, particularly those of clay minerals, vary continuously, leading to variations in mineral-OM associations.^27^ These variations, in turn, result in alterations of OM occurrence and impact the hydrocarbon generation patterns during diagenesis.^28,29^ Berthonneau et al. investigated the mineral-OM associations in shale with varying degrees of illitization and found that the organoclay complexes were dominated when smectite was abundant.^30^ However, as the illitization proceeded, OM gradually desorbed from clay minerals and became discrete particles. Du et al. conducted a comparative study on the hydrocarbon generation of various OM occurrences and found that the physically aggregated OM could participate in the hydrocarbon generation process at low temperatures but showed low hydrocarbon generation potential.^28^ In contrast, the mineral-adsorbed OM showed high thermal stability and converted into hydrocarbons when it desorbed.^31^ The hydrocarbon generation potential of the latter was high and contributed considerable amounts of the saturates and gaseous hydrocarbons.^32,33^ Consequently, the diversity in hydrocarbon generation mechanisms of OM with different occurrences during diagenesis appears to exert a profound influence on the enrichment of shale oil. Therefore, a comprehensive investigation into the heterogeneity of organoclay complexes is imperative for gaining insights into the accumulation patterns of shale oil.

Significant disparities exist in the hydrocarbon generation and petroleum distribution among shale intervals in the Dongying Depression.^34−36^ Despite the traditional hydrocarbon generation theory successfully explaining the varying characteristics and patterns of oil and gas resources, challenges remain in accounting for the discovery of deep-buried reservoirs, as well as the higher petroleum resources in shale formed in saline environments. To address these challenges, the present study collected shale samples from three intervals within the Dongying Depression and extracted the organoclay complexes. Using X-ray diffraction (XRD), Fourier transform infrared (FTIR) spectroscopy, thermogravimetric analysis (TGA), and Rock Eval VI pyrolysis, we aim to gain a comprehensive understanding of the mineral composition and OM occurrence in the organoclay complexes, as well as the impact to the shale oil formation. The results are expected to offer new insights into the complexities of shale oil accumulation from the perspective of mineral-OM interactions.

## 2 Materials and Methods

### 2.1 Samples

**Figure 1:**
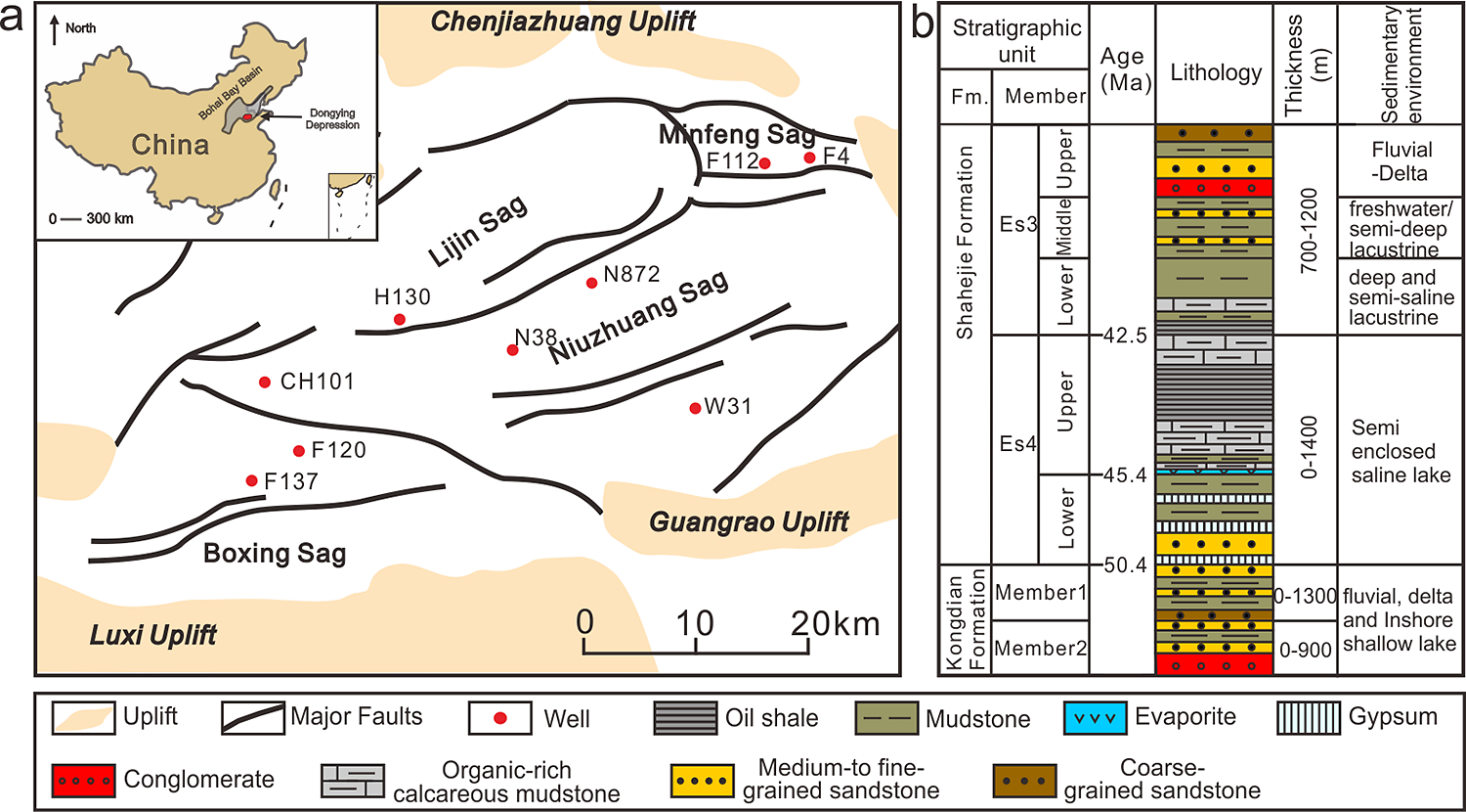
(a) Structure map of Dongying Depression and the location of sampling wells; (b) stratigraphic map of the Shahejie Formation. Adapted with permission from Zeng et al.^14^ Copyright 2022, the authors.

Twenty-two shale samples were collected from the Paleogene Shahejie Formation (Es) in the Dongying depression (Figure 1a), with a sampling depth of 2245–3492 m (Table 1). These samples were from three submembers of the Es, namely, the upper part of the Fourth Member (Es4s), and lower part of the Third Member (Es3x), and the middle part of the Third Member (Es3z). The depositional environment of the three intervals varied significantly (Figure 1b). The Es4s was deposited in a shallow-semi-deep and saline lacustrine environment. The Es3x were deposited in a deep and semisaline lacustrine environment., while the Es3z were deposited in freshwater and semideep lacustrine environment.

**Table 1:** Information about the Samples

sample no.	well	depth (m)	interval	lithology
1	H130	2245	Es3z	gray mudstone
2	Ch101	2291	Es3z	dark gray mudstone
3	Ch101	2357	Es3z	gray mudstone
4	F4	2704	Es3z	dark gray mudstone
5	H130	2787	Es3z	dark gray mudstone
6	H130	2882	Es3z	dark gray mudstone
7	F112	2905	Es3z	massive mudstone
8	H147	3032	Es3z	dark gray mudstone
9	H147	3042	Es3z	dark gray and lime mudstone
10	N872	3050	Es3z	dark gray mudstone
11	F137	2849	Es3x	dark gray calcareous mudstone
12	F112	3117	Es3x	lamellar mudstone
13	F112	3122	Es3x	lamellar mudstone
14	H130	3222	Es3x	dark gray shale
15	N872	3331	Es3x	brown shale
16	F137	3178	Es4s	gray mudstone
17	F120	3278	Es4s	dark gray and silty limestone
18	F120	3283	Es4s	dark gray and silty limestone
19	F112	3337	Es4s	lamellar mudstone
20	F120	3339	Es4s	dark gray and silty limestone
21	F112	3430	Es4s	gypsiferous mudstone
22	F112	3492	Es4s	gypsiferous mudstone

### 2.2 Methods

The shale samples were ground into powders using a hammer mill. Then, the clay-sized fractions (<2 μm in diameter) were collected via the Stokes sedimentation method for subsequent analyses of the organoclay complexes. The XRD analyses were conducted using a D/Max-RA diffractometer (Rigaku, Japan) with Cu*Kα* radiation at 40 Kv and 20 mA. The XRD patterns (3–30°2θ) of the oriented slides were recorded after being air-dried, ethylene-glycol saturated, and heated (250 and 550 °C). The randomly oriented slides were scanned from 3 to 40°2θ. The FTIR was employed using a Nicolet 6700 spectrometer (ThermoFisher Scientific, Waltham, Massachusetts, USA) to obtain the organic and mineral vibrations in the range of 4000–400 cm^–1^. The spectra of clay-sized fractions heated at 105, 250, and 550 °C were collected. The TGA analyses were conducted with a TGA/DSC1/1000 (METTLER TOLEDO, Swiss) analyzer. About 10 mg samples were heated from room temperature to 800 °C at a heating rate of 20 °C/min under a nitrogen atmosphere. The Rock Eval VI pyrolysis analyzer (Vinci Technologies, France) was used to measure organic variables, including free hydrocarbon S1 (mg/g), pyrolytic hydrocarbon S2 (mg/g), total organic carbon (TOC) content, hydrogen index (HI), oxygen index (OI), and Tmax (°C).

## 3 Results and Discussion

### 3.1 Differences in Mineralogy and Organic Characteristics of Organoclay Complexes

**Figure 2:**
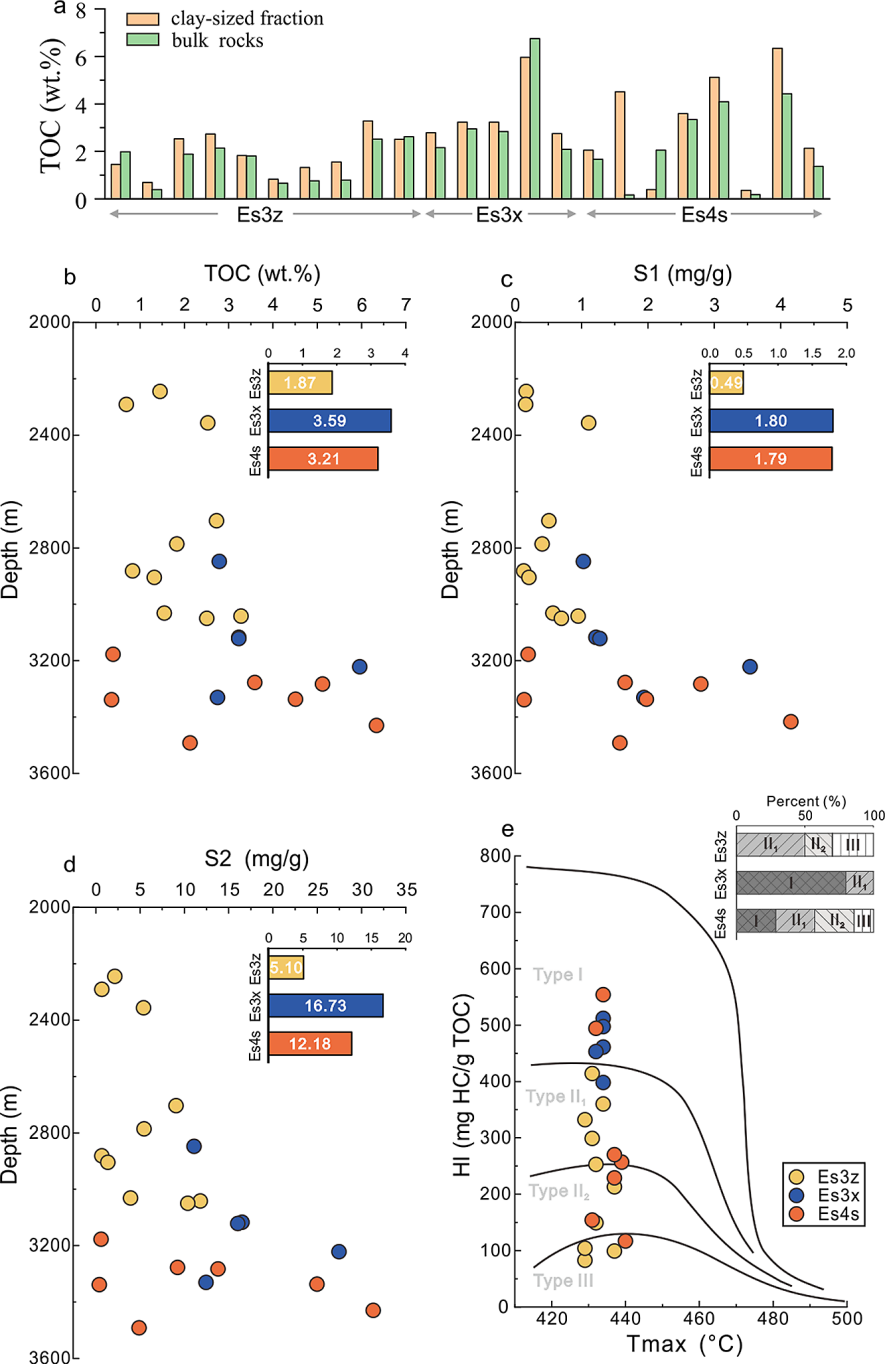
Organic characteristics of organoclay complexes in three intervals. (a) Comparison of TOC of the bulk rocks and corresponding clay-sized fractions. The variations in TOC (b), S1 (c), and S2 (d) with increasing depth. (e) OM type determined according to the hydrogen index (HI) and Tmax. The inset showed the average values of TOC, S1, S2, and OM type in the three intervals.

For the majority of the samples, the TOC of the organoclay complexes was higher than that of the corresponding bulk rocks, especially for samples with deep burial depths where the gap could reach as high as 2%, indicating the enrichment of OM in the organoclay complexes (Figure 2a). This suggests that organoclay complexes are the main contributors to the hydrocarbon generation within shale. The organoclay complexes exhibited high hydrocarbon generation potential and belonged to the excellent oil-generating source rocks, with most of their TOC (∼80%), S1 (∼45%), and S2 (∼50%) values exceeding 1%, 1 mg/g, and 10 mg/g, respectively. However, the TOC, S1, and S2 values in the organoclay complexes varied significantly in these three intervals, as indicated by the gradually elevated TOC, S1, and S2 values with increasing depth (Figure 2b–d). The HI-Tmax diagram indicated that the OM type also varied obviously (Figure 2e). Specifically, the organoclay complexes in Es3z were characterized by the lowest TOC, S1, and S2 values (1.87%, 0.49 mg/g, and 5.10 mg/g, respectively) and mainly composed of type II OM with a small amount of type III OM, indicating the poorest hydrocarbon generation potential. The organoclay complexes in Es3x, in which type I OM prevailed over type II OM, contained the highest TOC, S1, and S2 values (3.59%, 1.80 mg/g, and 16.73 mg/g, respectively) and exhibited the best hydrocarbon generation potential. The content of TOC, S1, and S2 (3.21%, 1.79 mg/g, and 12.18 mg/g, respectively) and hydrocarbon generation potential of Es4s organoclay complexes were between those of the other two intervals, with nearly equal amounts of type I, II, and III OM.

**Figure 3:**
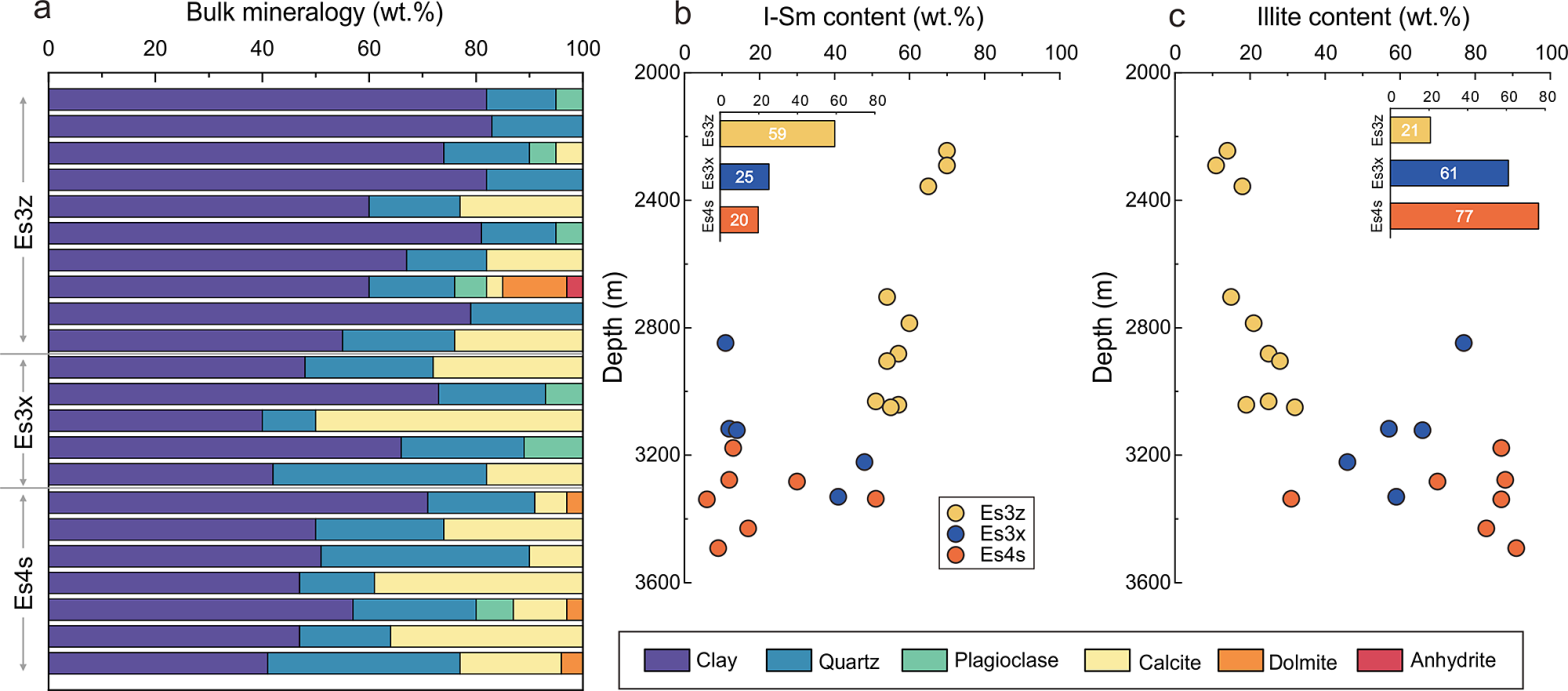
(a) Bulk mineralogy of the organoclay complexes. Variation in the content of I–Sm (b) and illite (c) with increasing depth. The inset showed the average smectite and illite content in the three intervals.

In addition, a notable difference also lied in the mineral composition of organoclay complexes among the three intervals. The bulk mineralogy was primarily composed of clay mineral (average 62%), quartz (average 21%), and calcite (14%) (Figure 3a). Plagioclase, dolomite, and anhydrite were only found in a few samples. As depth increased, the content of clay minerals gradually decreased, while the content of quartz and calcite significantly increased. Therefore, compared to the organoclay complexes in Es3z, the clay mineral content in Es3x and Es4s reduced to around 50% while those of quartz and calcite increased to around 24% and 20%, respectively.

The clay minerals primarily consist of illite-smectite mixed layers (I–Sm) and illite. As depth increased, the content of I–Sm significantly diminished while that of illite increased (Figure 3b,c). Specifically, the I–Sm contents in Es3z, Es3x, and Es4s were 59, 25, and 20%, respectively, while illite contents were 21, 61, and 77%, respectively. In general, the Es3z was dominated by I–Sm while Es3x and Es4s were dominated by illite. These variations in the clay mineralogy indicated that the smectite gradually converted into illite during diagenesis. In the process of smectite illitization, the modification of clay mineral structure would result in the release of Si, Al, and Ca into the pore fluid, which subsequently reprecipitated to form quartz, feldspar, and calcite.^37^ Therefore, the overall decrease in clay minerals and the concurrent increase in quartz and calcite in the bulk mineralogy further supported the conversion of smectite into illite.

**Figure 4:**
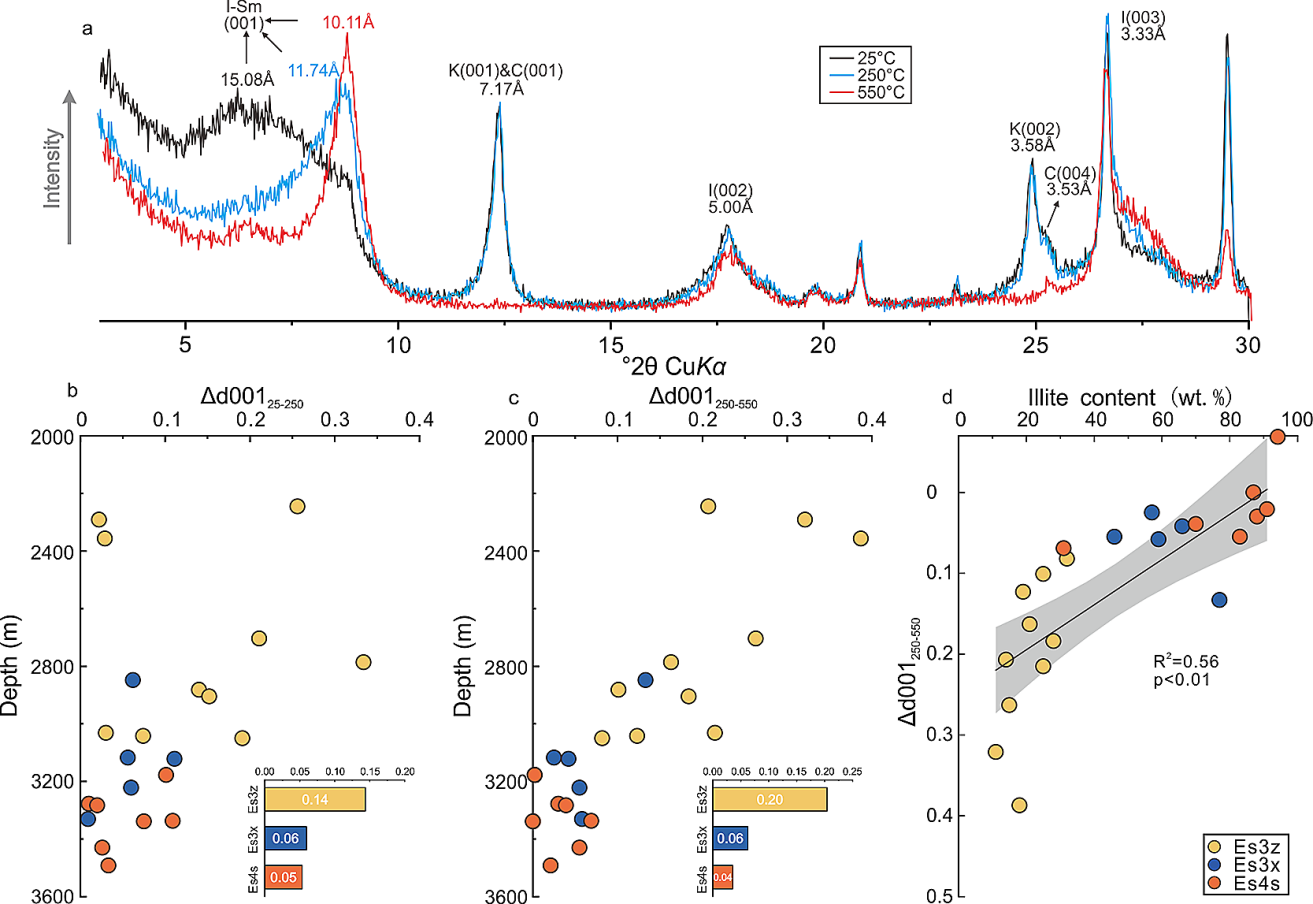
(a) Thermo-XRD patterns of the organoclay complexes; (b) variations in difference of d001 values between air-dried and 250 °C-heated organoclay complexes (Δd001_25–250_) with increasing burial depth; (c) variations in difference of d001 values between 250 °C-heated and 550 °C-heated organoclay complexes (Δd001_250–550_) with increasing burial depth; and (d) correlation between Δd001_250–550_ and the illite content.

**Figure 5:**
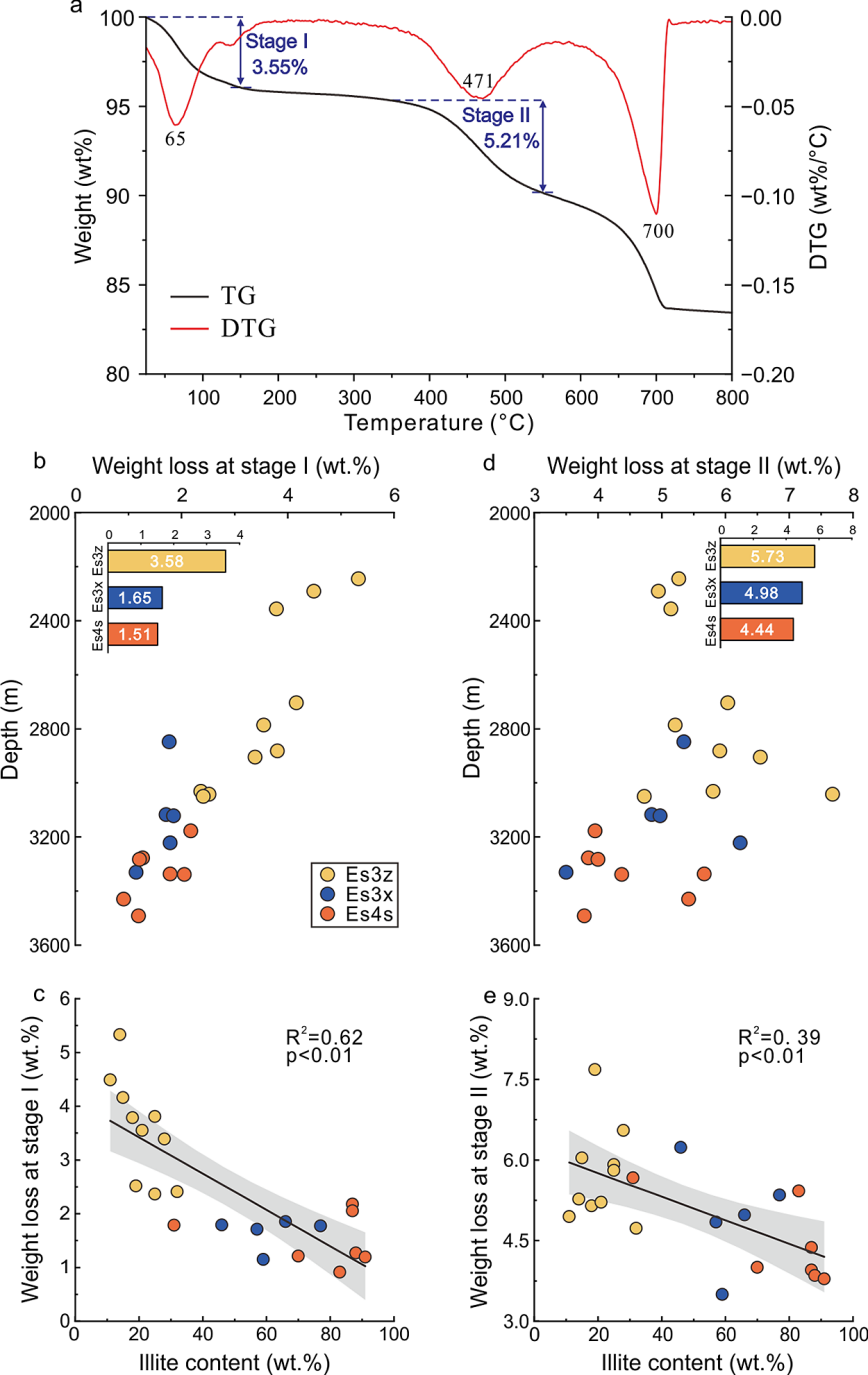
(a) TG and DTG curves. The stage I and stage II correspond to the mass losses of interlayer water and OM, respectively; (b) variations of mass loss in stage I with increasing burial depth. The inset showed the average values in the three intervals; (c) correlation between mass loss in stage I and illite content; (d) variations of mass loss in stage II with increasing burial depth. The inset showed the average values in the three intervals; (e) correlation between mass loss in stage II and illite content.

Furthermore, because smectite contains more interlayer water than illite, the smectite illitization process is normally accompanied by the reduction of interlayer water.^38^ The majority of interlayer water would be expelled when the organoclay complexes are heated at 250 °C;^39^ therefore, the difference of the d001 values between the air-dried and 250 °C-heated XRD patterns (Δd001_25–250_) can serve as a proxy for the interlayer water content (Figure 4a). The results revealed that the Δd001_25–250_ gradually decreased with increasing depth (Figure 4b), indicating a corresponding decline in interlayer water. Additionally, TGA analysis also revealed that the mass loss of interlayer water prior to 150 °C (stage I) progressively decreased as depth increased (Figure 5a,b). Specifically, the mass losses of the organoclay complexes in Es3z, Es3x, and Es4s in this stage were 3.58, 1.65, and 1.51%, respectively. Notably, the mass loss exhibited a significant negative correlation with the illite content (*R*^2^ = 0.62, *p* < 0.01) (Figure 5c). The variations in interlayer water content indicated the obvious smectite illitization process during diagenesis. In summary, the different diagenetic degrees of the three intervals led to distinct mineralogical characteristics.

To conclude, the organoclay complexes of the three intervals exhibited notable disparities in terms of OM composition, hydrocarbon generation potential, and mineral transformation degree. With increasing depth, the pyrolysis parameters, which are often used to reflect the hydrocarbon generation capacity, indicate that the Es3x organoclay complexes appear to be superior source rocks compared to the Es4s organoclay complexes (Figure 2). However, the practical exploration points to the contrary and has revealed that the proven oil and gas reserves from Es4s are significantly higher than those from the Es3x.^40,41^ Moreover, the molecular geochemical characteristics of crude oil also suggested a higher contribution from Es4s compared to Es3x.^42^ This contradiction arises because the hydrocarbon generation of source rocks is not solely controlled by variations in OM properties, such as types and maturity. Rather, the protection and catalysis provided by clay minerals also play an important role in determining the hydrocarbon generation potential.^28,30^ These effects are intimately linked to the properties of the clay minerals. Considering the elevated mineral transformation degree with increasing depth, it is necessary to account for the impact of mineralogical variations on hydrocarbon generation in different intervals, particularly the effects of varying clay-OM interactions attributed to the mineral transformation.

### 3.2 Different OM Occurrence in Organoclay Complexes

Pyrolysis experiments have revealed that the hydrocarbon generation potential of OM varied notably depending on the occurrence.^28^ Therefore, a thorough examination of the OM occurrence is the key point in comprehending the disparities in hydrocarbon generation of organoclay complexes.

**Figure 6:**
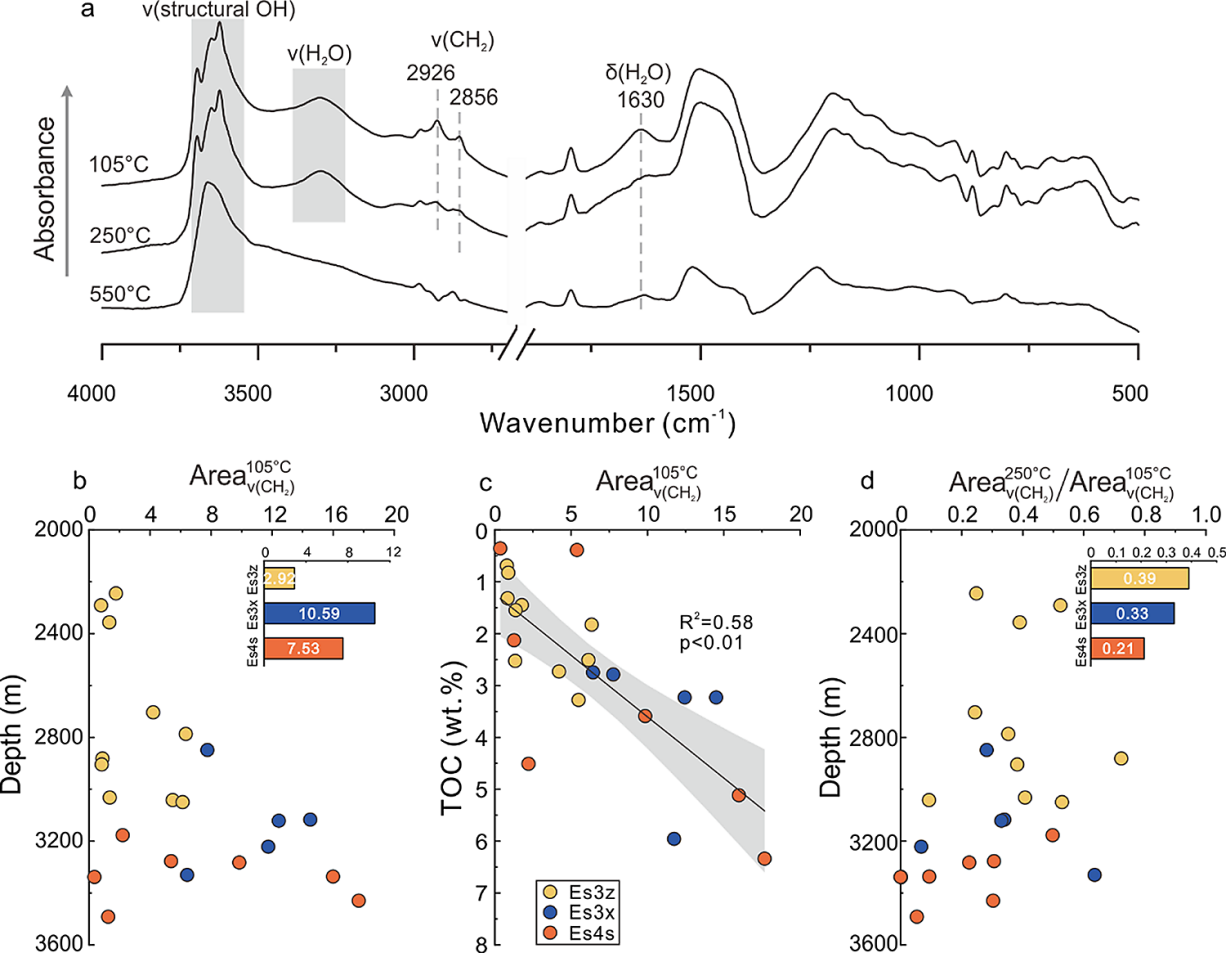
(a) Thermo-FTIR spectra of organoclay complexes; (b) variations of ν(CH_2_) area at 105 °C (Area_ν(CH_2_)_^105 °C^) with increasing burial depth. The inset showed the average values of Area_ν(CH_2_)_^105 °C^ in the three intervals; (c) correlation between Area_ν(CH_2_)_^105 °C^ and TOC; (d) ratio of ν(CH_2_) area at 250 and 105 °C (Area_ν(CH_2_)_^250 °C^/Area_ν(CH_2_)_^105 °C^) with increasing burial depth. The inset showed the average values of Area_ν(CH_2_)_^250 °C^/Area_ν(CH_2_)_^105 °C^ in the three intervals.

The vibrations of −CH_2_ (ν(CH_2_)) at 2926 and 2856 cm^–1^ indicated the presence of OM in the organoclay complexes (Figure 6a). The peak area of these two vibrations increased progressively with increasing burial depth (Figure 6b) and was significantly positive with the TOC (*R^2^* = 0.58, *p* < 0.01) (Figure 6c), indicating that the variations in the peak area could be used to represent those in the TOC. When the organoclay complexes were heated at 250 °C, the intensity of the ν(CH_2_) was obviously weaker compared to that heated at 105 °C (Figure 6a) although remained observable, indicating that partial OM within the organoclay complexes had been decomposed under 250 °C, which could be attributed to the presence of physically aggregated OM. Notably, the ν(CH_2_) completely disappeared after being heated at 550 °C, indicating the remained OM within the organoclay complexes has been completely decomposed and this portion of OM belonged to the chemically adsorbed OM. Therefore, the ratio of the ν(CH_2_) area at 250 °C to that at 105 °C (Area_ν(CH_2_)_^250 °C^/Area_ν(CH_2_)_^105 °C^) could be used to represent the proportion of chemically adsorbed OM to the total OM in the organoclay complexes. With increasing burial depth, this ratio gradually decreased, with values of 0.39, 0.33, and 0.21 in Es3z, Es3x, and Es4s, respectively (Figure 6d). This suggested that the proportion of chemically adsorbed OM gradually decreased during diagenesis.

Since the interlayer space of clay minerals provides crucial accommodations for OM, the variations in the d001 values observed in thermo-XRD analysis can provide valuable insights into the interlayer OM content.^39^ Under air-dried conditions, the d001 values typically range between 1.088 and 1.508 nm, generally exceeding 1.0 nm. After being heated at 250 °C, the d001 values significantly decreased to 1.000–1.316 nm and the 001 peaks were asymmetric on the low-angle side (Figure 4a). Heating at 250 °C resulted in the expulsion of interlayer water, leading to a reduction of d001 values. However, the d001 values higher than 1.0 nm and the observed asymmetry on the low-angle side suggested the presence of a more stable substance that cannot be expelled, namely, the interlayer OM. Upon heating at 550 °C, d001 values diminished to approximately 1.0 nm, and the 001 peaks were sharp and symmetric (Figure 4a). This indicated both the collapse of the interlayers and the release of the interlayer OM. Therefore, the difference of d001 values between 250 and 550 °C (Δd001_250–550_) could be used to characterize the variations of interlayer OM in the organoclay complexes. With the increasing burial depth, the Δd001_250–550_ gradually decreased, with values of 0.20, 0.06, and 0.03 nm for Ex3z, Es3x, and Es4s, respectively (Figure 4c). These results indicated that the interlayer OM was progressively released during diagenesis.

The TGA results showed that the mass loss before 250 °C (stage I) was slight except for the loss of adsorbed water, while an obvious mass loss was recognized from 350 to 550 °C (stage II) (Figure 5a). Due to the different thermal stability of physically aggregated OM and chemically adsorbed OM, the distinct mass loss in the two stages indicated that the latter was dominated in the organoclay complexes. Additionally, as the depth increased, the mass loss in stage II exhibited a consistent trend of gradual decrease, with specific values of 5.73% for Es3z, 4.98% for Es3x, and 4.44% for Es4s (Figure 5d).

The results of thermo-FTIR, thermo-XRD, and TGA all indicated that the OM occurrence evolved during diagenesis, with chemically adsorbed OM gradually decreasing. Meanwhile, both the Δd001_250–550_ and the mass loss in stage II exhibited a significant negative correlation with illite content (*R*^2^ = 0.56, *p* < 0.01 and *R*^2^ = 0.39, *p* < 0.01, Figures 4d and 5e), indicating that the variations in the OM occurrence were related to smectite illitization. When OM is chemically adsorbed on the clay mineral surface or intercalated into interlayers, the protection of clay minerals would enhance the stability of OM.^43^ As illitization proceeds, the decrease of the SSA and collapse of interlayer reduced the accommodation for OM adsorption, which ultimately led to the desorption of chemically adsorbed OM.^44^ In this way, the physically aggregated OM increased and the stability of the OM was reduced. Therefore, the illitization degree may play a key role in determining the OM occurrence in the organoclay complexes.

### 3.3 Heterogeneity of Organoclay Complexes Control the Shale Oil Formation

The coevolution of smectite illitization and OM occurrence indicated the remarkable mineral-OM interactions during diagenesis. Actually, the mineral-OM interactions initiate during the formation of organoclay complexes.^45,46^ In organoclay complexes, the OM content is normally correlated with the SSA of minerals, especially the smectite with expandable interlayers. The interlayers provide ample accommodation for OM, which enables the long-term stabilization of OM through the formation of organoclay complexes.^43^ As such, smectite serves as a primary host for the preservation of OM,^12^ regulating the temporal and spatial variations of OM in black shale.^47^ The enrichment of OM in the organoclay observed in this study (Figure 2), together with previous findings,^28,48^ strongly supports the assertion that organoclay complexes are the main contributor of hydrocarbon in the source rocks. Recently, Berthonneau et al. compared the hydrocarbon generation of source rocks with different maturities and proposed that the smectite-to-illite transformation triggers OM desorption and promotes OM maturation.^30^ Du et al. further found that the hydrocarbon generation of organoclay complexes is not solely controlled by thermal effects.^28^ Rather, the variations in mineral-OM interaction mechanisms during the smectite illitization process also impact the hydrocarbon generation patterns of organoclay complexes, leading to staged variations in the yield and composition of hydrocarbons.

**Figure 7:**
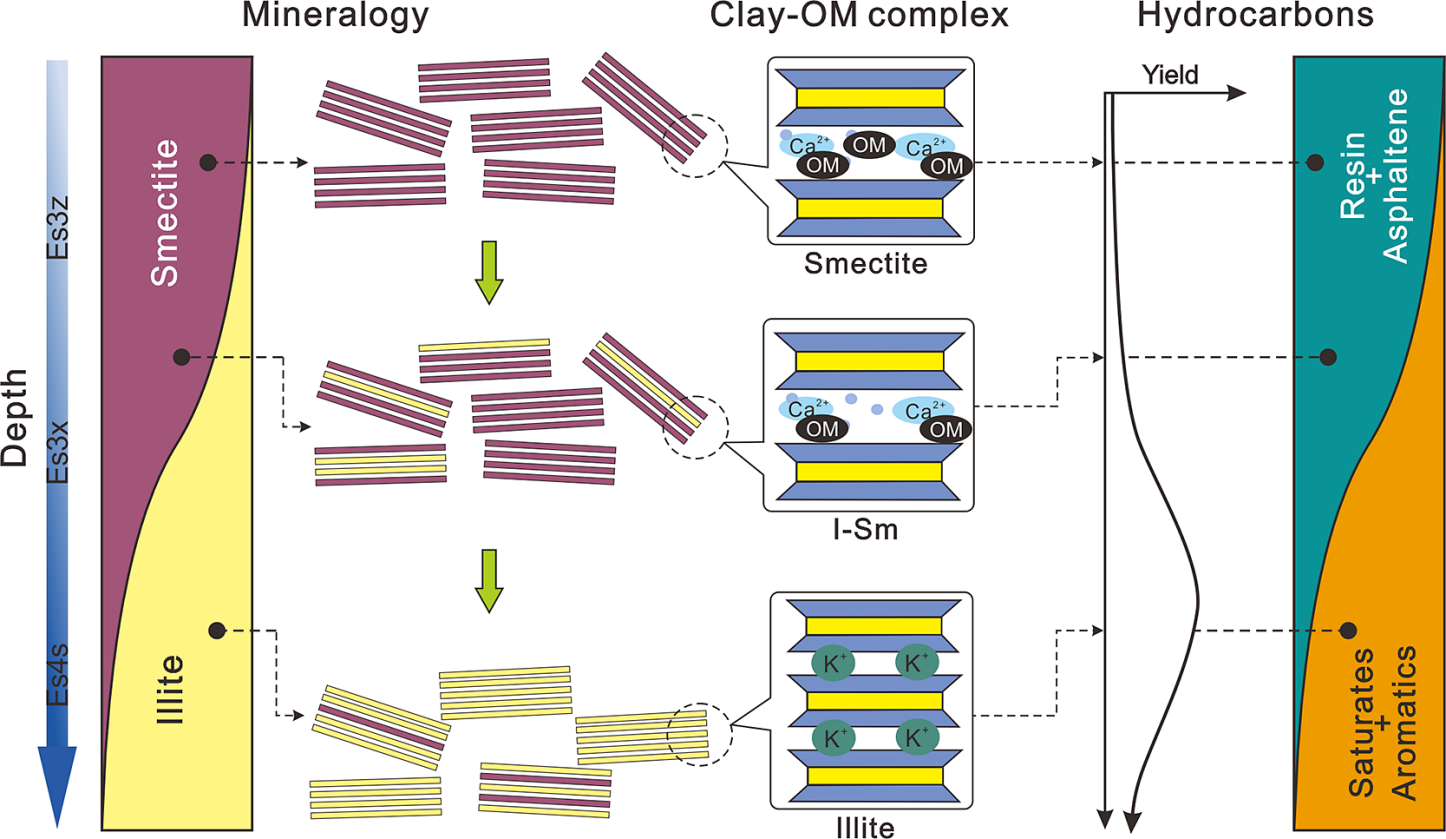
Comprehensive diagram of mineral transformation degree, variations in OM occurrence, characteristics of shale oil from different intervals. The yield and composition of shale oil are adapted with permission from Du et al.^49^ Copyright 2021, Elsevier.

In this study, when the smectite illitization degree was low in Es3z and Es3x, OM was primarily preserved within the interlayer of clay minerals. Despite the high OM content in the organoclay complexes, the protection of clay minerals effectively shied OM from thermal degradation. Consequently, the activation energy required for the hydrocarbon generation is elevated, leading to reduced shale oil production (Figure 7). Our previous research studies examining the composition of shale oil in Es3z and Es3x showed that the yield was low and primarily composed of resin.^49^ The low conversion of OM aligned with the finding that OM remained preserved within the interlayer of clay minerals in these two intervals. However, when the smectite illitization degree increased in Es4s, the illitization promoted the desorption of interlayer OM.^50^ On the one hand, the OM lost the protection from clay minerals, thereby facilitating further conversion of OM and boosting shale oil yield,^31^ leading to a peak in hydrocarbon generation in this interval. On the other hand, as the burial depth increased, the conversion of clay minerals would donate hydrogens and/or promote free radical formation of OM, thereby accelerating the hydrocracking and decarboxylation reactions, respectively. Therefore, high-degree clay mineral transformation in Es4s is regarded to promote the conversion of OM into low-molecular-weight hydrocarbons, such as saturates, aromatics, and gaseous hydrocarbons.^28^ This could explain the large amount of shale oil, which was primarily composed of saturates, observed in Es4s.

It should be noted that during the hydrocarbon generation, the high-quality OM (such as amorphous OM with high HI and low OI) would preferentially transform into hydrocarbons.^27^ As the hydrocarbon generation proceeds, the low-quality OM with low HI and high OI, which did not participate in hydrocarbon generation, would gradually accumulate within the organoclay complexes. Therefore, it can be inferred that OM in the Es3x has not undergone significant conversion into hydrocarbons, thereby exhibiting high OM content and maintaining high hydrocarbon generation potential. However, most of the OM in the organoclay complexes in Es4s has already been transformed into hydrocarbons under the effects of mineral catalysis, resulting in a decrease in chemically adsorbed OM content and a decline of the hydrocarbon generation potential.^51,52^ From this perspective, the yield and composition of shale oil would be decoupled with the pyrolysis parameters (e.g., TOC, S1, S2, HI, and OI). As the primary contributor to hydrocarbon generation, variations in mineral transformation and OM occurrence within organoclay complexes provide an explanation for the variations in yield and composition of shale oil, suggesting that the difference in organoclay complexes is the key factor regulating shale oil formation.

## 4 Conclusions

Remarkable heterogeneity of organoclay complexes in shale was found during diagenesis, primarily relating to the variations in mineral composition and OM occurrence. Specifically, the mineral transformation was obviously observed with increasing burial depth, especially the gradually elevated smectite illitization degree. Simultaneously, the OM types became worse, which was accompanied by the reduction of chemically adsorbed OM. In brief, the shale in shallow intervals exhibited a low degree of mineral transformation and was abundant in mineral-adsorbed OM, whereas the deep shale was characterized by a high mineral transformation degree and low mineral-adsorbed OM.

By comparing the evolution of clay mineral transformation and OM occurrence within organic-clay complexes with the previously published shale oil production and composition in the same formation, we found that the heterogeneity of organoclay complexes plays a predominant role in determining the yield and composition of shale oil, of course, the influence of the OM content and type cannot be denied. When the degree of mineral transformation is low, the OM remains trapped within the clay mineral interlayers, effectively preventing its conversion into hydrocarbons and resulting in low shale oil generation with the predominance of resin. However, as the burial depth increased, the elevated mineral transformation degree promoted the desorption of OM and its conversion into hydrocarbons, contributing to the high yield of shale oil with the predominance of saturates. This conclusion is particularly pertinent to argillaceous source rocks, where OM and clay minerals associate with each other during the formation of source rocks in the form of organic-clay complexes. In such scenarios, the heterogeneity of organoclay complexes emerges as a crucial factor in elucidating the generation and accumulation mechanisms of shale oil, providing insights into the determining factors of shale oil production and quality.
